# Risk factors for bile duct injury after laparoscopic cholecystectomy: A protocol for systematic review and meta-analysis

**DOI:** 10.1097/MD.0000000000028191

**Published:** 2021-12-10

**Authors:** Chang-Cheng Dong, Xue-Jun Jiang, Xue-Ying Shi, Bing Li, Liang Chen

**Affiliations:** a Department of General Surgery, Inner Mongolia Baogang Hospital (The Third Affiliated Hospital of Inner Mongolia Medical University), Kundulun District, Baotou, Inner Mongolia Autonomous Region, China; b Department of Hematology, Inner Mongolia Baogang Hospital (The Third Affiliated Hospital of Inner Mongolia Medical University), Kundulun District, Baotou, Inner Mongolia Autonomous Region, China.

**Keywords:** bile duct injury, bile fistula, cholecystectomy, laparoscopy, meta-analysis, risk factors

## Abstract

**Background:**

Bile duct injury (BDI) is one of the serious complications in laparoscopic cholecystectomy (LC), but there is currently a lack of systematic review of risk factors related to BDI after LC. This study conducts meta-analysis on the risk factors related to bile duct injury after LC, the purpose is to provide reference basis for preventing and reducing BDI after LC.

**Methods:**

Using the Computer to retrieve of Chinese and English databases such as CNKI, WANFANG Data, the VIP Network, PubMed, Embase, the Cochrane Library, etc. The time is from the establishment of each database until August 2021. A case–control study is selected that is related to the risk factors of BDI after LC. This meta-analysis using RevMan 5.4 and State 12.0 software is performed after two researchers independently sift through the literature, extract the data, and evaluate the bias risk included in the study.

**Results:**

The risk factors related to BDI after LC will be analyzed by systematic review.

**Conclusion:**

The conclusion of this study will play an important role in reducing BDI after LC.

**OSF Registration:**

DOI 10.17605/OSF.IO/2B3K9, the registration URL is https://osf.io/2b3k9.

## 1 Introduction

Laparoscopic cholecystectomy (LC) is a common method for treating gallbladder diseases, with the advantages of small trauma, simple surgical operation, short surgical time, fast recovery after surgery, etc.^[1,2]^ Bile duct injury (BDI) is a kind of serious and difficult surgical complications, and these complex injuries are often caused by LC, the incidence rate can reach 0.3% to 1.4%.^[3,4]^ The associated BDI after LC may lead to biliary peritonitis, bile duct stenosis, obstructive jaundice, biliary tract infection, and even more serious complications such as biliary cirrhosis, portal hypertension, liver atrophy, etc, which can increase the patient's pain, affect their postoperative recovery, and even endanger the patient's life and safety.^[5–7]^ Therefore, it is of great significance to analyze the risk factors that affect the related to BDI after LC and to formulate effective prevention and treatment strategies according to it. There are many literatures on the risk factors related to BDI after LC, but there are defects such as low sample size and incomplete risk factor indicators, so the significance of guiding clinical is limited.^[8,9]^ This study will conduct meta-analysis of the case–control study of the risk factors related to BDI after LC, aiming to screen out the risk factors and associated strength related to BDI after LC, and provide evidence-based medical evidence support for clinical prevention and reduction related to BDI after LC.

## 2 Methods

### 2.1 OSF registration number

The study has been registered on Open Science Framework (OSF), registration number: DOI 10.17605/OSF.IO/2B3K9 (website: https://osf.io/2b3k9). All steps of this study will be carried out in accordance with the requirements of the Preferred Reporting Items for Systematic Review and Meta-Analysis Protocols (PRISMA-P) 2015 guidelines.

### 2.2 Ethics and communication

This type of study is systematic reviews, and the entire study process does not involve the privacy information of individual patients, therefore does not require ethical approval.

### 2.3 Eligibility criteria

#### 2.3.1 Types of studies

A case-control study on the risk factors related to BDI after LC is collected by computer retrieval of professional databases. The language of the retrieval literature is set to Chinese and English.

#### 2.3.2 Types of patients

Patients who underwent BDI after LC. The patient's age, gender, course of disease, race, and so on are not limited.

#### 2.3.3 Inclusion criteria

1. Literature with original data.
2. The literature content is the risk factors related to BDI after LC.
3. The study design is a case–control study.

#### 2.3.4 Exclusion criteria

1. The research type does not meet the requirements.
2. Studies with incomplete clinical outcome data.
3. The format of the literature is review, abstract, letter, expert opinion, and case report.

### 2.4 Data sources and search strategies

Using the Computer to retrieve of Chinese and English databases such as CNKI, WANFANG Data, the VIP Network, PubMed, Embase, the Cochrane Library, etc. The time is from the establishment of each database until August 2021. To collect a case-control study on the risk factors related to BDI after LC. The search is carried out by a combination of subject terms and keywords.

### 2.5 Data extraction

The literature is independently screened, extracted, and cross-checked by two researchers. If there are differences, they are resolved through discussion or consultation with third parties. The literature is first selected to read the title, after excluding the obviously irrelevant literature, further read the abstract and the full text to determine whether to include. If necessary, contacting the original study authors by mail or phone for information that is not identified but is important for this study. The data extraction includes:

1. basic information incorporated into the study: research topics, first authors, published journals, etc.
2. baseline characteristics and interventions of the subjects.
3. key elements of bias risk assessment.
4. outcome indicators and result measurement data of concern.

**Figure 1:**
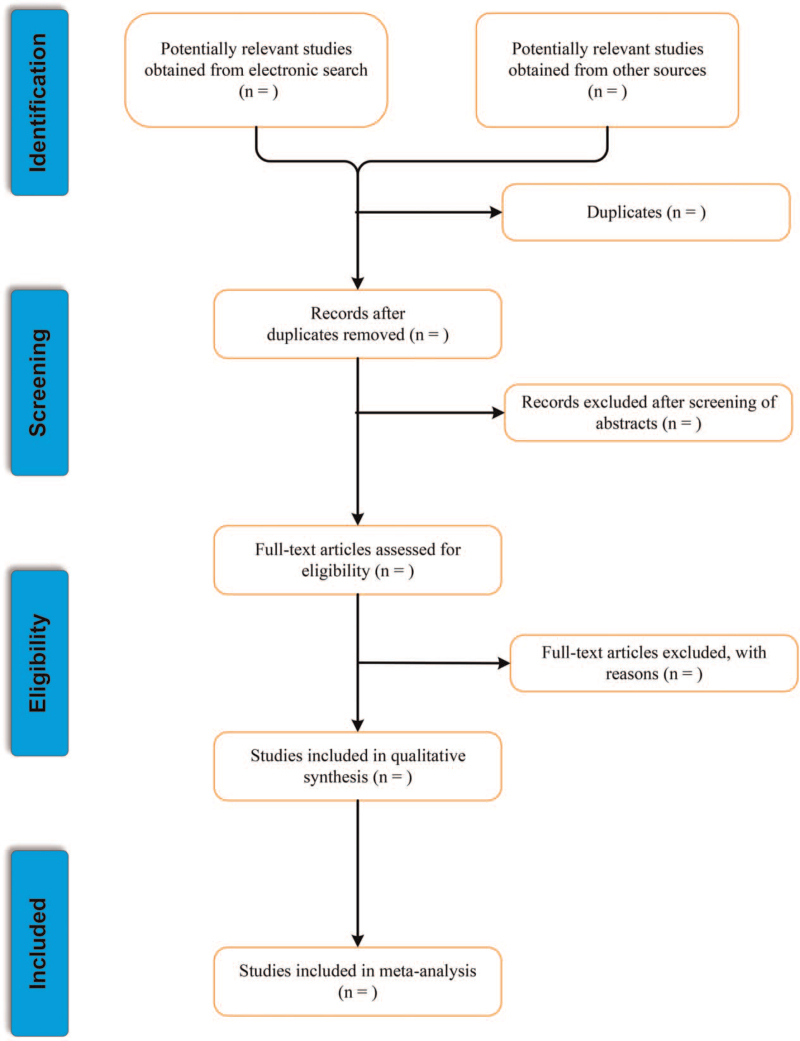
The PRISMA flow chart of the selection process.

The PRISMA flow chart of the selection process is shown in Figure 1.

### 2.6 Assessment of study quality

Two researchers independently evaluate the bias risk in the included study and cross check the results. In the case–control study, Newcastle Ottawa scale (NOS) is used to evaluate the quality of the included literature, with a full score of 9; high quality studies are classified as NOS score ≥ 7, and low-quality literature is classified as NOS score ≤ 6.^[10]^

### 2.7 Data extraction and meta-analysis

RevMan 5.3 software is used for data analysis. The heterogeneity test among the included studies adopts the *Q* test, *I*^2^ stands for heterogeneity: if *P* > .1 and *I*^2^ ≤ 50%, it means that it is statistically homogeneous, and the fixed-effects model is used for Meta-analysis; if *P* ≤ .1 and *I*^2^ > 50%, it means that there is statistical heterogeneity, and the random effects model is used. The odds ratio is used to represent the count data, and the weighted mean difference is used to represent the measurement data; the 95% confidence interval is calculated. Combined effect size test: If *P* ≤ .05, the difference is statistically significant. Obvious clinical heterogeneity is processed by subgroup analysis or sensitivity analysis, or only descriptive analysis.

### 2.8 Sensitivity analysis

This study mainly uses the research method of removing individual items for sensitivity analysis.^[11]^

### 2.9 Publication bias

Stata 12.0 software is used to select the visual method (funnel plot method) and statistical method (Egger method test) to evaluate the publication bias for the outcome indicators with the number of research documents ≥7.^[12]^

## 3 Discussion

Due to the continuous change of people's living and eating habits, the incidence of benign gallbladder diseases such as gallbladder stones and gallbladder polyps has gradually increased in recent years.^[13]^ LC is the “gold standard” procedure to treat the benign gallbladder disease. With the extensive development of LC in hospitals at all levels, the incidence rate of BDI has gradually increased in recent years.^[14]^ Therefore, it is of great significance to clarify the high-risk factors related to BDI after LC, formulate effective prevention and treatment strategies according to them, and reduce the occurrence of related BDI after LC, so as to promote postoperative recovery and improve prognosis.^[15]^ There are many domestic studies on high-risk factors related to BDI after LC. But there are different degrees of difference in the types of high-risk factors and the intensity of association. There is a lack of evidence-based medicine. It is necessary to conduct a comprehensive systematic evaluation of the relevant high-risk factors, in order to guide clinical practice, which is more patients who receive more benefit from LC.

## Author contributions

**Conceptualization:** Chang-Cheng Dong, Xue-Jun Jiang, Liang Chen.

**Data curation:** Chang-Cheng Dong, Xue-Jun Jiang, Xue-Ying Shi, Bing Li.

**Formal analysis:** Chang-Cheng Dong, Xue-Jun Jiang.

**Funding acquisition:** Liang Chen.

**Methodology:** Chang-Cheng Dong, Xue-Jun Jiang.

**Resources:** Chang-Cheng Dong, Xue-Jun Jiang, Xue-Ying Shi.

**Software:** Chang-Cheng Dong, Xue-Jun Jiang, Bing Li.

**Supervision:** Chang-Cheng Dong.

**Writing – original draft:** Chang-Cheng Dong, Xue-Jun Jiang, Xue-Ying Shi, Bing Li.

**Writing – review & editing:** Chang-Cheng Dong, Xue-Jun Jiang, Liang Chen.
